# Regulation of the tumour suppressor PDCD4 by miR-499 and miR-21 in oropharyngeal cancers

**DOI:** 10.1186/s12885-016-2109-4

**Published:** 2016-02-11

**Authors:** Xiaoying Zhang, Harriet Gee, Barbara Rose, C. Soon Lee, Jonathan Clark, Michael Elliott, Jennifer R. Gamble, Murray J. Cairns, Adrian Harris, Samantha Khoury, Nham Tran

**Affiliations:** The Sydney Head and Neck Cancer Institute, Chris O’Brien Lifehouse, Sydney, Australia; Department of Infectious Diseases and Immunology, University of Sydney, Sydney, NSW Australia; Cancer Research UK Molecular Oncology Laboratories, Weatherall Institute of Molecular Medicine, University of Oxford, Oxford, OX3 9DS UK; Discipline of Pathology, School of Medicine, University of Western Sydney and Cancer Pathology, Bosch Institute, University of Sydney, Sydney, Australia; Central Clinical School, University of Sydney, NSW, Australia; South Western Clinical School, University of NSW, Sydney, Australia; Centre for the Endothelium, Vascular Biology Program, Centenary Institute, Sydney, Australia; Schizophrenia Research Institute, Sydney, NSW Australia; School of Biomedical Sciences, Faculty of Health, and Hunter Medical Research Institute, University of Newcastle, Callaghan, NSW Australia; Centre of Health Technologies. Faculty of Engineering and Information Technology, University of Technology, NSW, Australia

## Abstract

**Background:**

The rates of oropharyngeal cancers such as tonsil cancers are increasing. The tumour suppressor protein Programmed Cell Death Protein 4 (PDCD4) has been implicated in the development of various human cancers and small RNAs such as microRNAs (miRNAs) can regulate its expression. However the exact regulation of PDCD4 by multiple miRNAs in oropharyngeal squamous cell carcinoma (SCC) is not well understood.

**Results:**

Using two independent oropharyngeal SCC cohorts with a focus on the tonsillar region, we identified a miRNA profile differentiating SCC tissue from normal. Both miR-21 and miR-499 were highly expressed in tonsil SCC tissues displaying a loss of PDCD4. Interestingly, expression of the miRNA machinery, Dicer1, Drosha, DDX5 (Dead Box Helicase 5) and DGCR8 (DiGeorge Syndrome Critical Region Gene 8) were all elevated by greater than 2 fold in the tonsil SCC tissue. The 3’UTR of PDCD4 contains three binding-sites for miR-499 and one for miR-21. Using a wild-type and truncated 3’UTR of PDCD4, we demonstrated that the initial suppression of PDCD4 was mediated by miR-21 whilst sustained suppression was mediated by miR-499. Moreover the single miR-21 site was able to elicit the same magnitude of suppression as the three miR-499 sites.

**Conclusion:**

This study describes the regulation of PDCD4 specifically in tonsil SCC by miR-499 and miR-21 and has documented the loss of PDCD4 in tonsil SCCs. These findings highlight the complex interplay between miRNAs and tumour suppressor gene regulation and suggest that PDCD4 loss may be an important step in tonsillar carcinogenesis.

**Electronic supplementary material:**

The online version of this article (doi:10.1186/s12885-016-2109-4) contains supplementary material, which is available to authorized users.

## Background

Cancers of the head and neck region commonly arise from the mucosal surfaces of the oral cavity, larynx and oropharynx. The incidence of head and neck squamous cell carcinoma (HNSCC) has increased gradually over the last 3 decades [1]. HNSCC is one of the top six malignancies affecting men worldwide [1] and the 6th leading cause of cancer mortality globally [2]; it is estimated that 500,000 new cases will arise this year. Despite improvements in clinical care, survival rates of approximately 50 % have remained unchanged for the past several decades [1]. According to the most recent NCI SEER database (http://seer.cancer.gov/faststats/selections.php?#Output), incidence rates for oropharynx and tonsil cancers have been increasing since the year 2000. Given these trends, we still have a limited understanding of molecular pathways which control the development of oropharyngeal SCCs, although the human papillomavirus is a known risk factor for tonsil cancers [3]. Gene array studies have identified potential cellular candidates for biomarkers, oncogenes and tumor suppressors [4, 5].

MicroRNAs regulate genes at the post-transcriptional level by binding to the target 3’ untranslated region (UTR) and promoting target gene cleavage or translational inhibition [6, 7]. These small RNAs are frequently deregulated in human malignancies such as the breast, lung, colon, and liver [8] and play a major role in tumorigenesis. Profiling studies of HNSCC cell lines [9] and HNSCCs [10, 11] have shown deregulation of miRNA expression. One of the key miRNAs frequently upregulated in human cancers, including HNSCCs, is miR-21 [10]. This miRNA [12] and others (for example miR-150 [13], miR-182 [14]) target the tumor suppressor programmed cell death protein 4 (PDCD4), which has been implicated in the development and progression of several human cancers [15]. Given the numerous miRNA-binding sites on the 3’UTR of PDCD4, it is likely that regulation is mediated by multiple miRNAs. Our study shows a unique focus on tonsillar-derived miRNAs and investigates the possible regulation of PDCD4 by multiple miRNAs.

## Methods

### Patient cohort

The cohort consisted of 43 patients (39 males, 4 females) treated for tonsillar cancer at Royal Prince Alfred Hospital Sydney, Australia between 2002 and 2006. The mean age was 57 years (range 39–80). Seventeen SCCs and matched microscopically normal adjacent (2 cm outside the surgical margin) tissues proved suitable for the profiling analyses. Fixed paraffin-embedded cancers from 36 of the 43 patients including the 17 used in profiling studies were used for PDCD4 immunohistochemistry (Additional file 2: Table S2). Investigations were approved by the Research Ethics Committee at Royal Prince Alfred Hospital, Sydney, Australia (Protocols X05-269, X05-270). This protocol covered consent and collection of material excess to diagnostic requirements for research purposes only. A second independent cohort sourced from the UK (*n*=18, 14 males, 4 females) had a median age of 63 (range 42–92,) was used for further miRNA validation. All UK patients gave written informed consent in accordance with the Helsinki Declaration of 1975, revised 2000. Ethical approval was obtained from the local institutional Research Ethics Boards ethics (approval #09/H0606/5, Oxford and South Manchester).

### Cell line culture and transfection

HNSCC cell lines used in this study included SCC089 [16], SCC003 (tonsil) [16], SCC099 (floor of mouth) [16] and SCC029b (oral cavity) [17] were kindly provided by Dr. Guy Lyons (Sydney Cancer Centre, Royal Prince Alfred Hospital, Australia). All HNSCC cell lines were HPV16 negative. HEK-293 cells were purchased from ATCC (USA). All cell lines were maintained in DMEM medium with 1 % L-glutamine (JRH Biosciences, USA) supplemented with 10 % fetal bovine serum (JRH BiosciencesTM, USA), and 100 mg/L penicillin/streptomycin (Life Technologies, USA) at 37 °C. Transfections were performed using Lipofectamine RNAiMAX (Life Technologies, USA) in triplicate and in 6-well plates. The Locked Nucleic Acid (LNA) miR-21 antisense and LNA negative control (Exiqon, Denmark), pre-miR-21, pre-miR-499 and pre-miR-negative control#1 (Life Technologies, USA) were separately or double transfected into 2.5 x 10^5^ cells per well to a final concentration of 30 pmol per well. For PDCD4, 100 ng of the vector was transfected in combination with the miRNAs at 30 pmol per well. Cells from replicate wells were combined, harvested and divided into two equal parts and stored at–70 °C until subsequent RNA and protein analysis. Transfection efficiency was determined by measuring the relative expression level of the miRNA using Quantitative Real Time-Polymerase Chain Reaction (QRT-PCR).

### Isolation of RNA from fresh tissue or cultured cells

Approximately 100 mg of fresh frozen tissue was diced homogenized and then rinsed with 1 ml of Trizol reagent (Invitrogen, USA). For the cell lines (SCC089, SCC003 and HEK-293), 1 ml of Trizol was added to the cell pellet with further disruption through a 21-gauge needle. Total RNA was then extracted using isopropanol precipitation and quantified using a NanoDrop ND 1000 (Thermo Fisher Scientific, USA). Samples with ratios of 260/280 in the range of 1.71 to 2.1 were used for the downstream studies.

### Total RNA labeling

MicroRNAs were labeled at 3’-end with a P-CU-C3-Cy3 RNA linker by RNA ligation as described [18]. A 60 μL ligation reaction was prepared with 6 μg of total RNA, 0.1 mM ATP, 20 mM MgCl_2_, 3.5 mM DTT, 10 mg/ml BSA, 10 % DMSO, 50 mM HEPES, pH 7.8, 250 ng of P-CU-C3-Cy3 (GeneLink, USA) and 20 units of T4 RNA ligase (NEB, USA). The reaction was incubated on ice for 2 h followed by precipitation at–70 °C for 20 min with 0.3 M sodium acetate, 0.5 mg/ml glycogen (Life Technologies, USA) and 2 volumes of 100 % ethanol to remove any unbound RNA-linkers. Each labeled sample was dissolved in 30 μl of 400-fold diluted ULS labeled reference set, then mixed with 300 μl Church and Gilbert hybridization buffer. This mixture was denatured at 95 °C for 2 min before hybridization. A mixture of 371 synthetic DNA reference oligonucleotides (Sigma-Genosys, Australia) containing complementary sequences to all LNA probes, was randomly labeled using the ULYSIS labeling kit (Invitrogen, USA) and then filtered using a MicroSpinTM G-25 column (Amersham, USA). Aliquots of a 400-fold dilution of labeled reference set were stored at–20 °C until needed.

### Microarrays

A commercial LNA-modified oligonucleotides library (Exiqon, Denmark) based on miRBase release 7.1, covering 371 human and mouse miRNA was utilized for expression profiling. Features were deposited onto GAPS II slides (Amersham, USA) at a concentration of 10 μM (Australian Genome Research Facility, Australia). Individual miRNA LNA probes were printed four times on each array. In addition, all samples were arrayed in technical duplicate. Pre-hybridization of array slide was performed in 3x SSC, 0.1 % SDS, 0.2 % BSA at 60 °C for 1 h. Each slide was then rinsed with in full DEPC treated water, followed by 100 % ethanol. Hybridization was then performed in disposable reaction chambers (ABGene, USA). The combined hybridization mixture of 330 μl was injected into the chamber, and then incubated in a hybridization oven with constant rotation at 5 rpm for 3 h at 52 °C. Slides were then washed briefly in 4x SSC, twice in 2x SSC plus 0.1 % SDS, twice in 0.2 % SSC and twice in 0.1 % SSC. These were then scanned with a Genepix 4000B Scanner (Axon Instruments, USA).

### Microarray data analysis

Raw data manipulation and downstream statistical analyses were performed using the TM4 suite (http://www.tm4.org) [19]. Normalization included Lowes correction followed by in-slide replicate analysis. The data were then filtered by a percentage cut-off of 95 % and then subjected to statistical data mining. Two-class un-paired hierarchical clustering (HCL) of samples was constructed with average linkage and Pearson correlation. Differential miRNA expression was then analyzed by Significance Analysis of Microarrays (SAM). A list of significantly expressed miRNA genes was generated with a false discovery rate (FDR) of 0. The data discussed in this publication have been deposited in NCBI's Gene Expression Omnibus [20] and are accessible through GEO Series accession number GSE75630 (http://www.ncbi.nlm.nih.gov/geo/query/acc.cgi?acc=GSE75630).

### Reverse transcription quantitative real-time PCR (QRT-PCR)

QRT-PCR setup and analysis were performed using the MIQE guidelines [21]. In brief, total RNAs were treated with RNase-free DNaseI (Promega, Australia). Measurement of individual miRNAs or genes was determined using a two-step QRT-PCR approach. cDNA was firstly generated using the Hi-capacity cDNA Reverse Transcription Kit (Life Technologies, USA). For QRT-PCR detection, we use TaqMan specific kits. The miRNA primers plus the U75 RNA primer were combined in equal molar concentrations into 500 ng of total RNA to generate miRNA cDNA. For genes such as Dicer, random hexamers were added to 500 ng of total RNA. A standard 15 μl reverse transcription reaction was performed with the end product diluted 1:4 for the subsequent QRT-PCR. All QRT-PCR reactions were performed in technical triplicate using the Universal PCR Master Mix, No AmpErase UNG (2x) (Life Technologies, USA) and cycled on a 7900 Real-Time PCR System (Life Technologies, USA). Mean C^t^ values were normalized using the nuclear RNA U75 for miRNA expression or B2M for normal genes. U75 was used as it represented a stable calibrator, which did not change between our patient’s samples. Similarly, we utilised B2M as the reference gene for normalisation of mRNA expression. Relative expression level of a given miRNA or gene was calculated using DeltaDelta Ct method and presented as fold change relative to the control [22].

### Cloning of the PDCD4 constructs

The PDCD4 coding sequence and the first 789 bp of the 3’UTR (NM-014456.3) containing the miR-21 and three miR-499 binding sites were chemically synthesized into the pJ246 vector (DNA 2.0 Inc, USA) with *EcoRI, EagI* sites at the 5’ start of the UTR and *NotI* at the 3’ end. The PDCD4 coding region with and without 709 bp 3’UTR fragment was then excised with *EcoRI/NotI* or *EcoRI/EagI* and sub-cloned into the MCS of the pCI-neo vector (Promega, Australia).

### Western blotting

Cells were lysed on ice in buffer containing 50 mM HEPES (pH 7.5), 150 mM NaCl, 10 % glycerol, 1 mM EGTA, 10 mM Na pyrophosphate, 100 mM NaF and 1 mM NaVO_4_. Protein concentration was determined by using the DC protein assay kit using BSA as a standard (BioRad, USA). Protein separation was achieved using 30 μg on the NuPAGE (4-12 %) Bis-Tris Gel (Invitrogen, USA) and then transferred onto PVDF membrane (PIERCE, USA). Membranes were blocked with 5 % skim milk for 1 h and then incubated with primary anti-PDCD4 rabbit antibody (Sigma-Aldrich, USA) at a 1: 1000 dilution overnight at 4 °C. The membrane was then washed with PBS-0.05 % Tween-20 and conjugated with the secondary IgG-HRP anti-rabbit (Amersham Biosciences, USA) in 1:10,000 for 1 h. Specific protein signals were detected using the ECL plus reagent (Amersham Biosciences, USA). Beta-actin was detected using a rabbit polyclonal at 1:5000 dilution (Abcam, USA) and secondary reaction with a goat polyclonal anti rabbit IgG, 1:2000 dilution (Abcam, USA).

### Immunohistochemistry

Sections were deparaffinized, rehydrated, antigen retrieved as previously described [23]. Slides were then incubated with a 1:300 anti PDCD4 rabbit Ab (Sigma-Aldrich, USA) for 1 h with subsequent detection using the EnVision + Dual Link System (Dako, USA). All reactions were conducted on the DAKO Autostainer, Universal Staining System (DAKO, USA). The staining was scored by three independent observers including one pathologist and classified as: (−) negative staining; low to high positive staining (+ to +++), and scored as percentage of stained cells [24]. The reduction of PDCD4 staining was determined by comparison of the PDCD4 staining between normal epithelial and tumor tissues in the same section.

## Results

**Fig. 1:**
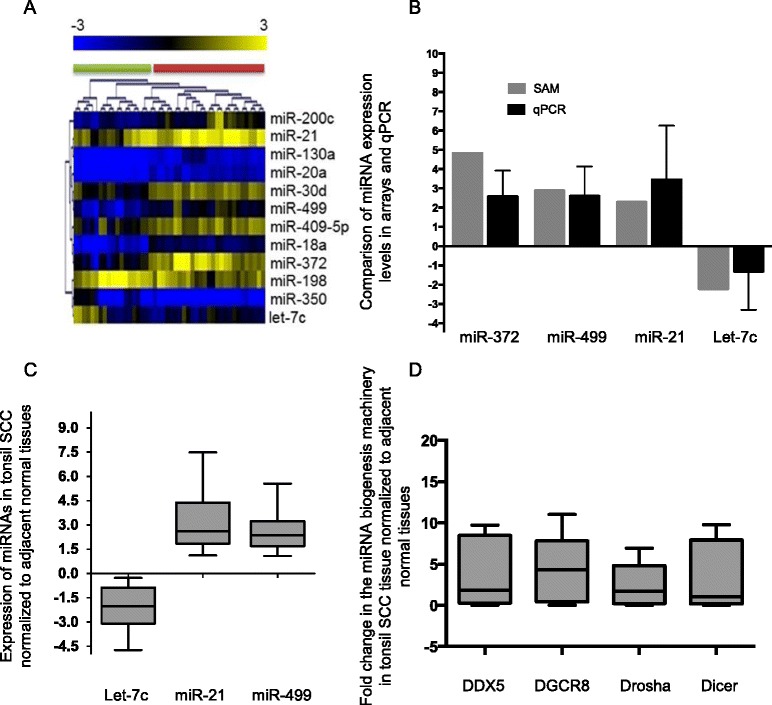
**a** Heatmap showing two class-SAM analyses for differentially expressed miRNAs in tonsil cancers. Green bar: normal samples, red bar: SCC samples. miRNA genes shown in yellow and blue represent up-regulated and down-regulated, respectively. **b** Validation of miR-372, miR-499, miR-21 and let7c in the Australian patient cohort (*n* = 10). Each miRNA was determined in the tumor tissue with fold expression normalized to paired normal tissue. **c** Validation of let-7c, miR-21 and miR-499 was performed using a second UK patient cohort. **d** Expression of the miRNA biogenesis machinery in 10 paired tonsil SCC samples. Expression of each gene was determined in the tumor tissue and then normalized to the paired normal tissue

Seventeen tonsillar cancers SCCs and matching adjacent macroscopically normal tissues were subjected to miRNA expression profiling. The data were mined using unsupervised hierarchical clustering (HCL) and principal component analysis (PCA) (Additional file 1: Figure S1). This indicated that tonsillar cancers had a distinct miRNA profile when compared to normal tissue and this signature was able to cluster samples in either a tumor or normal group. We identified differentially expressed miRNAs using the statistical analysis of microarrays (SAM) algorithm [25] (Fig. 1a). When we applied SAM analysis to include a 2-fold cut off, miR-350 was eliminated. In contrast, the eleven other miRNAs showed a 2-fold change in expression. This yielded eleven differentially expressed miRNAs, nine of which were upregulated while two miRNAs were downregulated in cancers relative to normal tissue (Table 1). Some of the upregulated miRNAs included, miR-499, miR-372, miR-18a, miR-21 and miR-30d, while let-7c and miR-198 were downregulated.

**Table 1:** (A) List of deregulated miRNAs identified in tonsillar cancers. These miRNAs demonstrate a two-fold change from normal tonsillar tissue. (B) ΔΔG free energy value, which can be used to evaluate target site accessibility. This value was determine using the PITA algorithm (http://genie.weizmann.ac.il/pubs/mir07/mir07_prediction.html) for the first 789 bp 3’UTR of PDCD4

MicroRNA	Fold change
miR-372	4.82
miR-499	2.89
miR-18a	2.82
miR-200c	2.69
miR-130a	2.59
miR-21	2.29
miR-30d	2.21
miR-409-5p	2.14
miR-20a	2.12
let-7c	0.45
miR-198	0.40

The array data were then confirmed by QRT-PCR of 4 representative miRNAs using ten tumour and adjacent normal samples. This indicated a strong concordance between the two data sets (Fig. 1b). Given that most of the miRNAs were upregulated, we also measured the expression of the biogenesis machinery using QRT-PCR. The analysis of ten tonsil SCC with paired normal tissue showed that Dicer1, Drosha, DDX5 and the DiGeorge critical region gene (DGCR8) were all upregulated by greater than two-fold relative to normal tissue (Fig. 1d). The expression levels of miR-21 and miR-499 are known to control the translation of the tumour suppressor PDCD4 and let-7 has been shown to regulate Dicer levels. Given the importance of these miRNAs we further confirmed their expression using an independent cohort of tonsillar specimens sourced from the UK (*n*=−22 tonsillar cancers and matched normal tissues) (Fig. 1c).

**Fig. 2:**
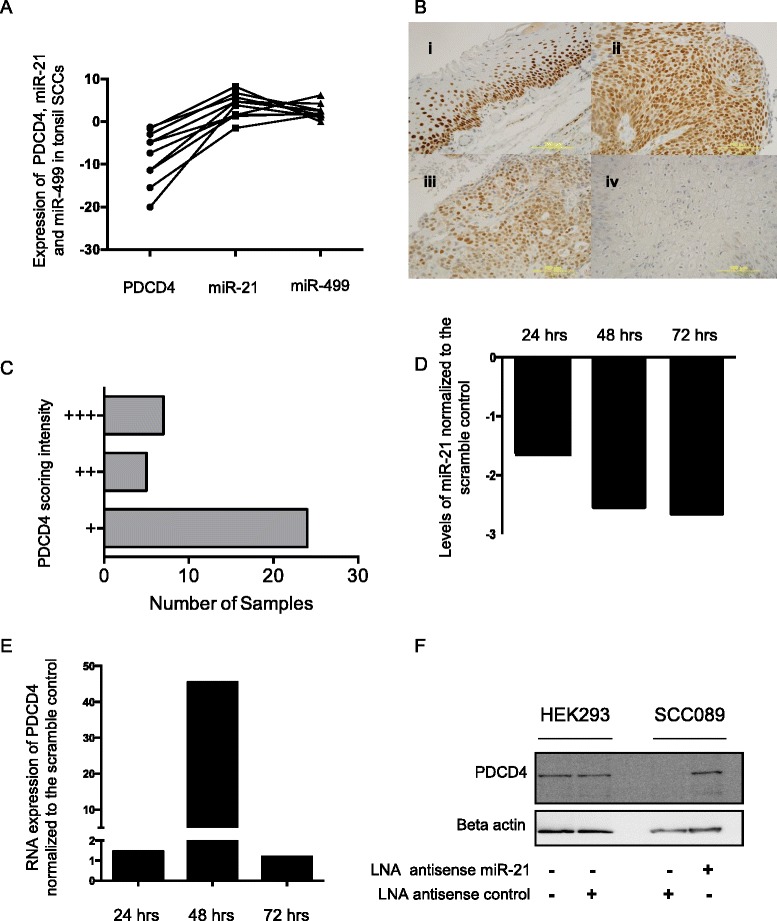
**a** Expression levels of PDCD4, miR-21 and miR-499 in tonsil SCC tissue normalized to expression in adjacent healthy tissue (10 patients were analyzed in this cohort). **b** Panel i) Representative staining of PDCD4 in normal epithelium with a 3+ scoring intensity. Panel ii) PDCD4 staining in Tonsil SCC tissue with a 3+ scoring intensity. Panel iii) PDCD4 staining in Tonsil SCC tissue with a 2+ scoring intensity. Panel iv) negative PDCD4 staining in Tonsil SCC tissue. **c** Summary of PDCD4 expression in tonsil cohort (+ to +++, with three + being high expression). **d** Reduction of miR-21 levels using a LNA anti-sense in SCC089 cell lines. Expression of miR-21 was then normalized to the LNA antisense scramble control. **e** PDCD4 mRNA levels in miR-21 depleted cells normalized against the LNA antisense scramble control. **f** Protein Expression of PDCD4 in miR-21 depleted SCC089 and HEK-293 cells

The 3’ UTR of PDCD4 contains three binding sites for miR-499 and one for miR-21 (Additional file 1: Figure S2). In tonsillar SCCs samples, both miR-21 and miR-499 were elevated but the RNA levels for PDCD4 was markedly lower (Fig. 2a). Furthermore, 30 of the 36 tonsil cancers examined by semi quantitative immunohistochemistry showed reduced expression of PDCD4 in tumour cells relative to the surrounding normal epithelial cells (Fig. 2b and c and Additional file 2: Table S2).

Four HNSCC cancer cell lines (SCC029b, SCC003, SCC099, SCC089) were screened by QRT-PCR to determine the relationship between miR-21 and PDCD4 expression (Additional file 1: Figure S3). In all the HNSCC cell lines, there was an inverse relationship between the levels of miR-21 and PDCD4 expression. Given SCC089 cells showed high endogenous levels of miR-21 and could be easily transfected, we delivered a LNA antisense to regulate expression of miR-21. At 48 h and 72 h post transfection, miR-21 levels were reduced by greater than two-fold (Fig. 2d). The reduction in miR-21 was marked by a large increase in PDCD4 mRNA and protein expression at 48 h in SCC089 cells (Fig. 2e and f respectively). In addition, the LNA antisense control showed no effect on PDCD4 expression in either control HEK-293 cells or SCC089 cells. This effect was also confirmed using another independent HNSCC cell line (SCC003- Additional file 1: Figure S4). Therefore the reduction of miR-21 levels in tonsillar cancer cell lines was marked by an increase in PDCD4 expression.

**Fig. 3:**
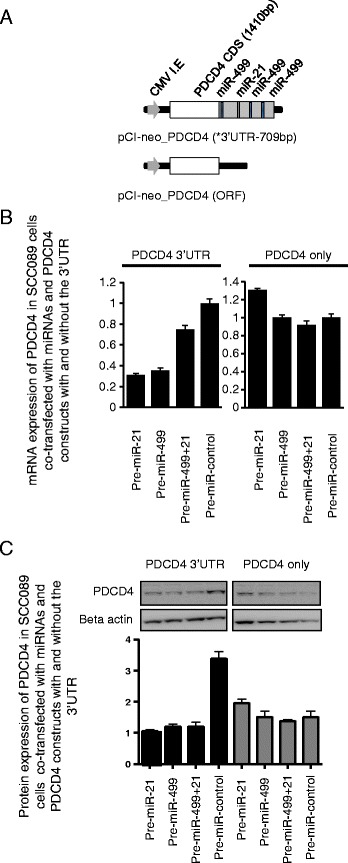
**a** Schematic representation of the PDCD4 constructs with and without the 3’UTR. **b** mRNA expression of PDCD4 in SCC089 cells transfected with PDCD4 constructs with and without the 3’UTR. These cells were also co-transfected with either miR-21, miR-499 alone or in combination and harvested 24 h post transfection. PDCD4 levels were only normalized to the reference gene B2M and fold change calculated using DeltaDelta Ct method. **c** Protein expression of PDCD4 in the co-transfected cell described in (B). PDCD4 expression was then quantitated relative to Beta-actin levels

To investigate whether miR-21 and miR-499 directly interact with the 3’UTR of PDCD4, the open reading frame (ORF) of PDCD4 with and without a 789 bp section of its 3’UTR which contained the miR-21 and miR-499 sites was cloned into the pCI-neo expression vector (Fig. 3a). Given SCC089 cells showed low endogenous levels of PDCD4, these cells were transiently transfected to overexpress PDCD4 with and without the 3’UTR in combination with miR-21 or miR-499. We observed no downregulation of PDCD4 lacking its 3’UTR. In contrast, when the 3’UTR was included, PDCD4 mRNA expression was significantly reduced by miR-21 and miR-499 (Fig. 3b). Similarly, PDCD4 protein expression was significantly downregulated only in cells with the PDCD4 3’UTR (Fig. 3c). We also transiently transfected SCC003 cells with PDCD4 with and without the 3’UTR, and as expected, PDCD4 mRNA expression was downregulated by both miR-21 and miR-499 only when the 3’UTR was present (Additional file 1: Figure S5).

**Fig. 4:**
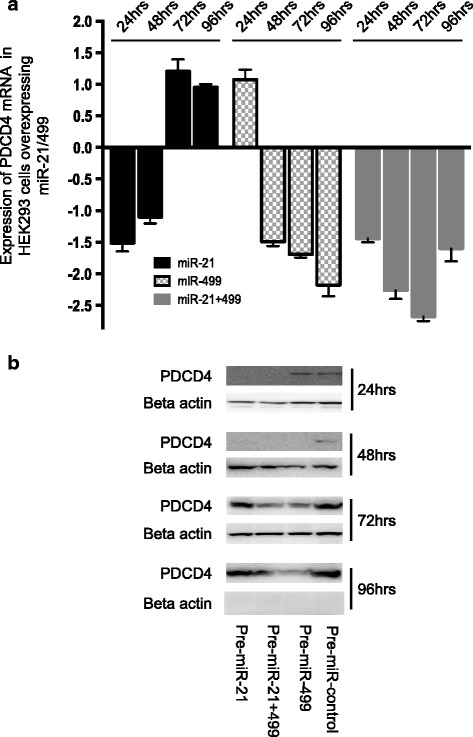
**a** Expression of PDCD4 mRNA in HEK-293 cells overexpressing miR-21 or miR-499 alone or in combination. PDCD4-mRNA fold change values were expressed relative to control cells containing the Pre-miR control and calculated using the DeltaDelta Ct method for *n* = 3. **b** Protein expression of PDCD4 in the same HEK-293 cells described above. PDCD4 protein expression was reduced by miR-21 at 24 and 48 h but returns to basal levels by 72 and 96 h. In contrast, miR-499 decreases PDCD4 expression from 48 h thereafter. As expected the combination of both miRNAs reduce PDCD4 mRNA and protein expression from 24 h

The PDCD4 3’UTR has over 31 broadly conserved miRNA sites. Thus, it may be plausible that both miR-21 and miR-499 can regulate PDCD4. Using the HEK-293-cell line, which expresses stable levels of PDCD4, we transfected, miR-21/miR-499 alone or in combination and levels of PDCD4 were measured over a 96 h time period. Cells overexpressing miR-21 showed a reduction in PDCD4 mRNA and protein at only 24 and 48 h and not the later time points (Fig. 4a and b). In contrast, miR-499 had no affect at 24 h with suppression of PDCD4 only seen at 48 h and being sustained for the duration of the time course. As expected, cells containing both miRNAs demonstrated reduced expression of PDCD4 throughout the entire time course. Furthermore, we measured the expression of the transfected miRNAs and this indicated consistent high expression of both miR-21 and miR-499 at all-time points (Additional file 1: Figure S6A and B). The exact conditions were then tested in the SCC003 tonsillar cells with similar findings (Additional file 1: Figure S6C). Taken together these observations suggest that miR-21 and miR-499 can both regulate the expression of PDCD4.

## Discussion

Our study identified eleven differentially expressed miRNAs in a series of tonsillar cancers, nine of which showed greater than a two-fold change. In line with previous reports of head and neck cancer [10, 11, 26–34] miR-499, miR-372, miR-18a and miR-21 were upregulated in our series. The upregulation of miR-30d seems unique to our analysis. The downregulation of let-7c and miR-198 is supported by other recent findings [10, 28]. Several of these miRNAs were validated using an Australian and UK tonsillar SCC cohorts further supporting their clinical expression. That is, the miRNAs expression is robust and display similar expression levels in different tonsil SCC patients.

As the majority of miRNAs were elevated compared to normal tissue, this may suggest an alteration in the miRNA biogenesis machinery. Subsequent QRT-PCR measurement of Drosha, Dicer1, DDX5 and DGCR8 indicated a general overexpression of all four biogenesis components in tonsillar cancers. These findings may be linked with the downregulation of let-7, which has been shown to negatively regulate Dicer1 expression [35]. The upregulation of Dicer1 in tonsillar cancers is consistent with studies in salivary pleomorphic adenomas [33] and oral cancers [36]. These findings may suggest that deregulation in the biogenesis machinery is linked to the deregulation of specific miRNAs in tonsillar cancers. Indeed this may be a wider phenomenon as other cancers such as bladder [37], prostate [38] and lung adenocarcinomas [39] also demonstrate aberrant expression of either Dicer1 or Drosha.

To understand the regulatory role of miRNAs in tonsillar carcinogenesis we investigated PDCD4 as a target. PDCD4 is a tumor suppressor [40] and an inhibitor of protein translation [41]. Little is known about the expression and regulation of this tumor suppressor in tonsillar cancers. We show that miR-21 and miR-499 directly interact with the 3’UTR of PDCD4 in both HEK-293 and tonsil cancer cell lines. The single miR-21 site was able to elicit the same magnitude of suppression as the three miR-499 sites. There was also no significant additive suppressive effect in cells overexpressing both miR-21 and miR-499. Importantly, staining for PDCD4 demonstrated a loss of expression which is similar to the profile observed in lung [42], colon [43], prostate [43], and ovarian cancers [44]. In these cancers, loss of PDCD4 expression was associated with disease progression. It is not known whether PDCD4 expression is a predictor of outcome in tonsil cancer. However reduction of PDCD4 was marginally associated with nodal metastasis in oral cancers [45].

Reis et al. showed that PDCD4 is regulated by miR-21 in head and neck cancers [45]. More recently, miR-499 was shown to also regulate PDCD4 in colorectal cancer [46]. Our study has extended these findings by showing that in tonsillar SCCs, PDCD4 is also regulated by miR-499 and miR-21. The initial suppression at 24 h appeared to be mediated by miR-21 only. However as the time course was extended, miR-21 was unable to suppress PDCD4. In contrast, miR-499 had no effect initially but was effective at the 48 h and beyond. The delay in miR-499 suppression at 24 h cannot be attributed to miR-499 levels, as overexpression of the mature miR-499 was similar at all-time intervals. Furthermore, expression of the synthetic mature miR-21 was consistent over the 96 h but suppression of PDCD4 was only apparent at 24 h.

This regulation of PDCD4 by miR-499 and miR-21 may be explained by target site accessibility and seed region constraints. Evidence now indicates that binding sites 15 nt from the stop codon and sites positioned in the centre of long UTR’s display reduced silencing ability [47]. One of the miR-499 sites is located within 13 nt of the stop codon, whilst the other two are placed closer to the centre of the UTR. In contrast, the miR-21 site is positioned outside these accessibility constraints and therefore may have the ability to exert a rapid silencing effect. We calculated site accessibility by determining the ΔΔG free energy value using the PITA model, Table 2 [48]. The miR-21 site showed a lower free energy value and is interpreted as being more accessible than the other miR-499 sites. Furthermore, the seed region of miR-21 is a 8mer match while the miR-499 seed is at best a 7mer-1A. Considering these factors, we propose that may be miRISC/miR-21 initially binds to the miR-21 site to rapidly mediate PDCD4 gene silencing within 24 h. After this initial binding, the miRISC/miR-21 may recruit other factors to expose the downstream miR-499 sites. These sites would now be accessible to miR-499, which would maintain the silencing of PDCD4. Although our model awaits further elucidation it does establish the exciting premise that miR-21 may induce gene repression by promoting the accessibility of other miRNA sites.

**Table 2:** (A) List of deregulated miRNAs identified in tonsillar cancers. These miRNAs demonstrate a two-fold change from normal tonsillar tissue. (B) ΔΔG free energy value, which can be used to evaluate target site accessibility. This value was determine using the PITA algorithm (http://genie.weizmann.ac.il/pubs/mir07/mir07_prediction.html) for the first 789 bp 3’UTR of PDCD4

microRNA	Position	ΔGduplex	ΔGopen	ΔG
miR-21	241	−10.8	−8.1	−2.69
miR-499	466	−9.5	−7.91	−1.58
miR-499	532	−17.6	−16.02	−1.57
miR-499	17	−15	−21.04	6.04

It must also be noted that our tumour samples and cell lines were HPV16 negative. Thus, the deregulation of miR-21 and miR-499 was not influenced by the presence of the virus. However, the study by Ko et al., did report miR-21 up regulation in 30 % of their HPV16 positive tumours [49]. We also conducted a separate profiling study (unpublished data), which investigated the miRNA profile between HPV16 positive and HPV16 negative tonsil SCC. We found that miR-21 and miR-499 were not up-regulated in HPV16 positive tonsil SCCs. Interestingly, the cohort of miRNAs were very different and we suggest that, HPV16 may control the expression of other specific miRNAs and which are not common to HPV negative tumours.

## Conclusions

In summary, this study has characterized the expression of miRNAs in tonsillar cancers using two independent patient cohorts. Some of these miRNAs appear to be unique for tonsillar cancers. This is very pertinent given the lack of reliable biomarkers for this disease. We have further shown that the tumor suppressor PDCD4 is lost in the majority of tonsil SCCs and PDCD4 is directly regulated by two miRNAs. There appears to be a novel interplay between miR-499 and miR-21 in the regulation of PDCD4. Although further studies are needed to dissect this interaction, these results do highlight the complexity of miRNA-target gene regulation and suggest that PDCD4 loss may be an important step in tonsillar carcinogenesis.

## Additional files

Additional file 1:
**Supporting Data.** (PPTX 861 kb) Additional file 2:
**Patient Data.** (PPTX 73 kb)

## Abbreviations

DDX5: dead box helicase 5
DGCR8: DiGeorge Syndrome Critical Region Gene 8
HNSCC: head and neck squamous cell carcinoma
LNA: locked nucleic acid
miRNAs: microRNAs
PDCD4: programmed cell death protein 4
QRT-PCR: quantitative real time-polymerase chain reaction
SAM: statistical analysis of microarrays
SCC: squamous cell carcinoma
UTR: untranslated region
