# Supplementary material for: Regulation of the tumour suppressor PDCD4 by miR-499 and miR-21 in oropharyngeal cancers
Source: BMC Cancer. 2016 Feb 11;16:86. doi: 10.1186/s12885-016-2109-4 (PMC4750294; doi:10.1186/s12885-016-2109-4)
Supplement: Additional file 1: — Supporting Data. (PPTX 861 kb) [file 12885_2016_2109_MOESM1_ESM.pptx]

## Slide 1
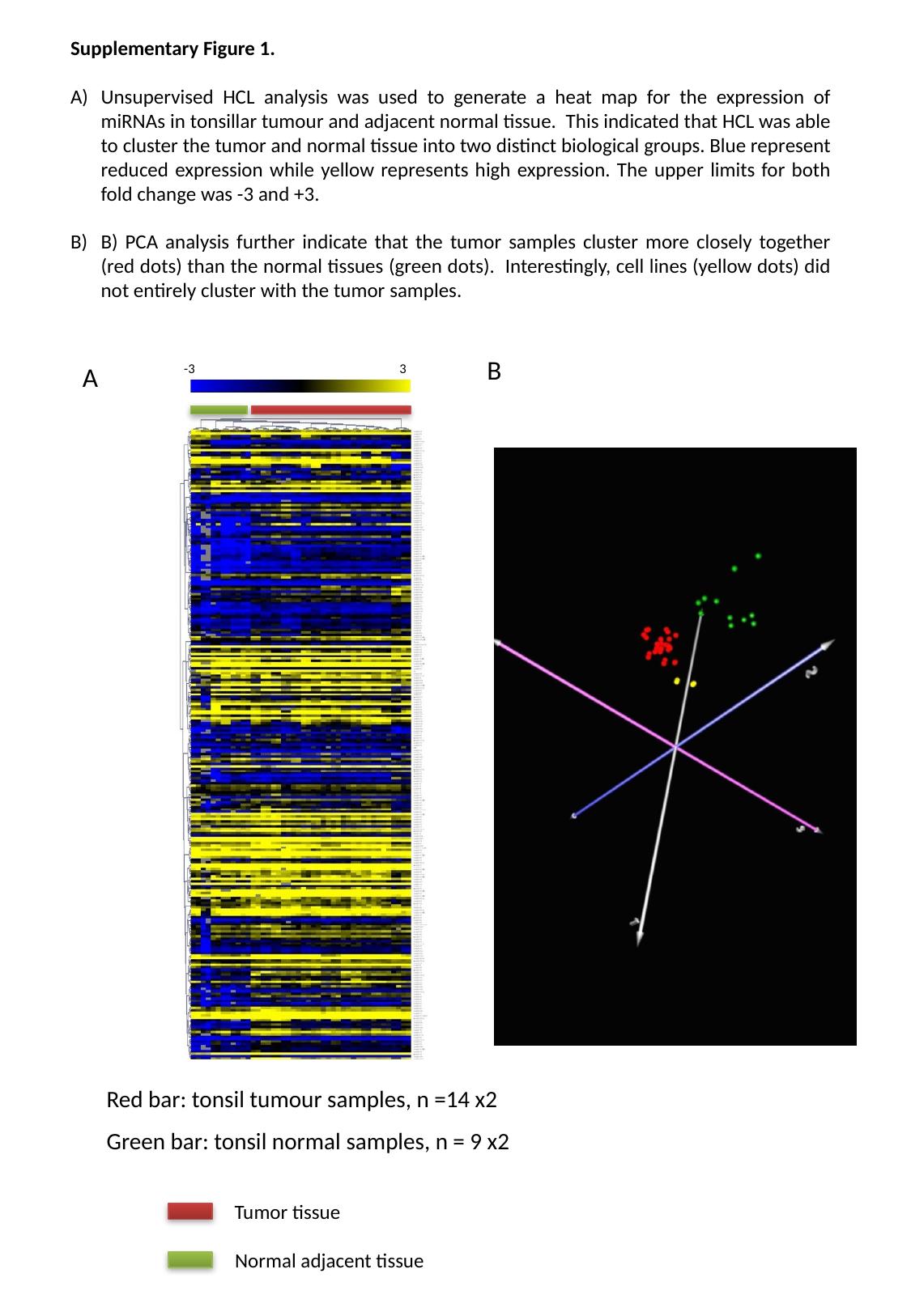

Supplementary Figure 1.
Unsupervised HCL analysis was used to generate a heat map for the expression of miRNAs in tonsillar tumour and adjacent normal tissue. This indicated that HCL was able to cluster the tumor and normal tissue into two distinct biological groups. Blue represent reduced expression while yellow represents high expression. The upper limits for both fold change was -3 and +3.
B) PCA analysis further indicate that the tumor samples cluster more closely together (red dots) than the normal tissues (green dots). Interestingly, cell lines (yellow dots) did not entirely cluster with the tumor samples.
B
-3
3
A
Red bar: tonsil tumour samples, n =14 x2
Green bar: tonsil normal samples, n = 9 x2
Tumor tissue
Normal adjacent tissue

## Slide 2
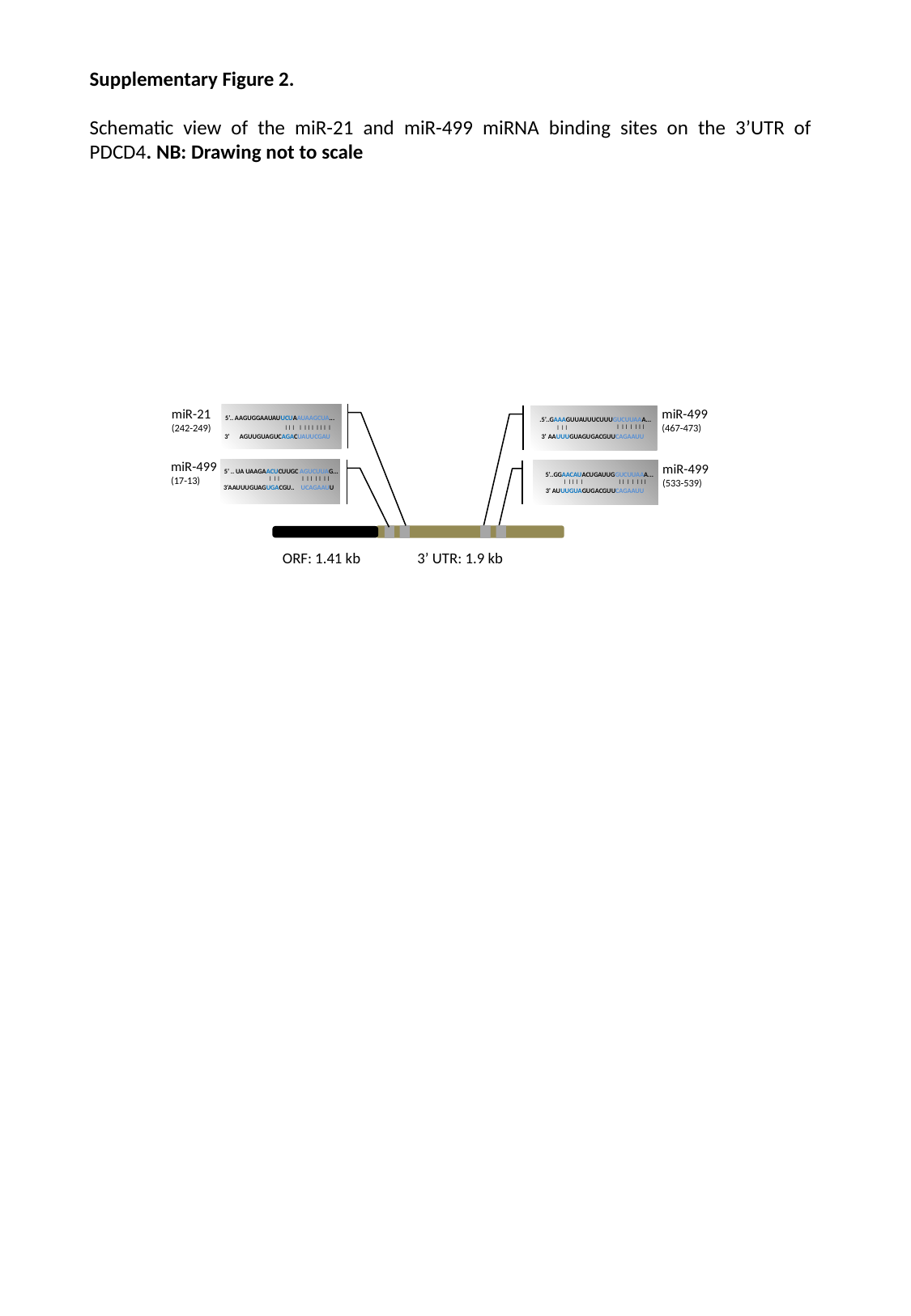

Supplementary Figure 2.
Schematic view of the miR-21 and miR-499 miRNA binding sites on the 3’UTR of PDCD4. NB: Drawing not to scale
miR-21
(242-249)
miR-499
(467-473)
5’.. AAGUGGAAUAUUCUAAUAAGCUA...
| | |
| | | | | | | |
3’ AGUUGUAGUCAGACUAUUCGAU
.5’..GAAAGUUAUUUCUUUGUCUUAAA...
| | | | | | |
| | |
 3’ AAUUUGUAGUGACGUUCAGAAUU
miR-499
(17-13)
miR-499
(533-539)
5’ .. UA UAAGAACUCUUGC AGUCUUAG...
| | |
| | | | | | |
UCAGAAUU
3‘AAUUUGUAGUGACGU..
5’..GGAACAUACUGAUUGGUCUUAAA...
| | | | |
| | | | | | |
 3’ AUUUGUAGUGACGUUCAGAAUU
ORF: 1.41 kb
3’ UTR: 1.9 kb

## Slide 3
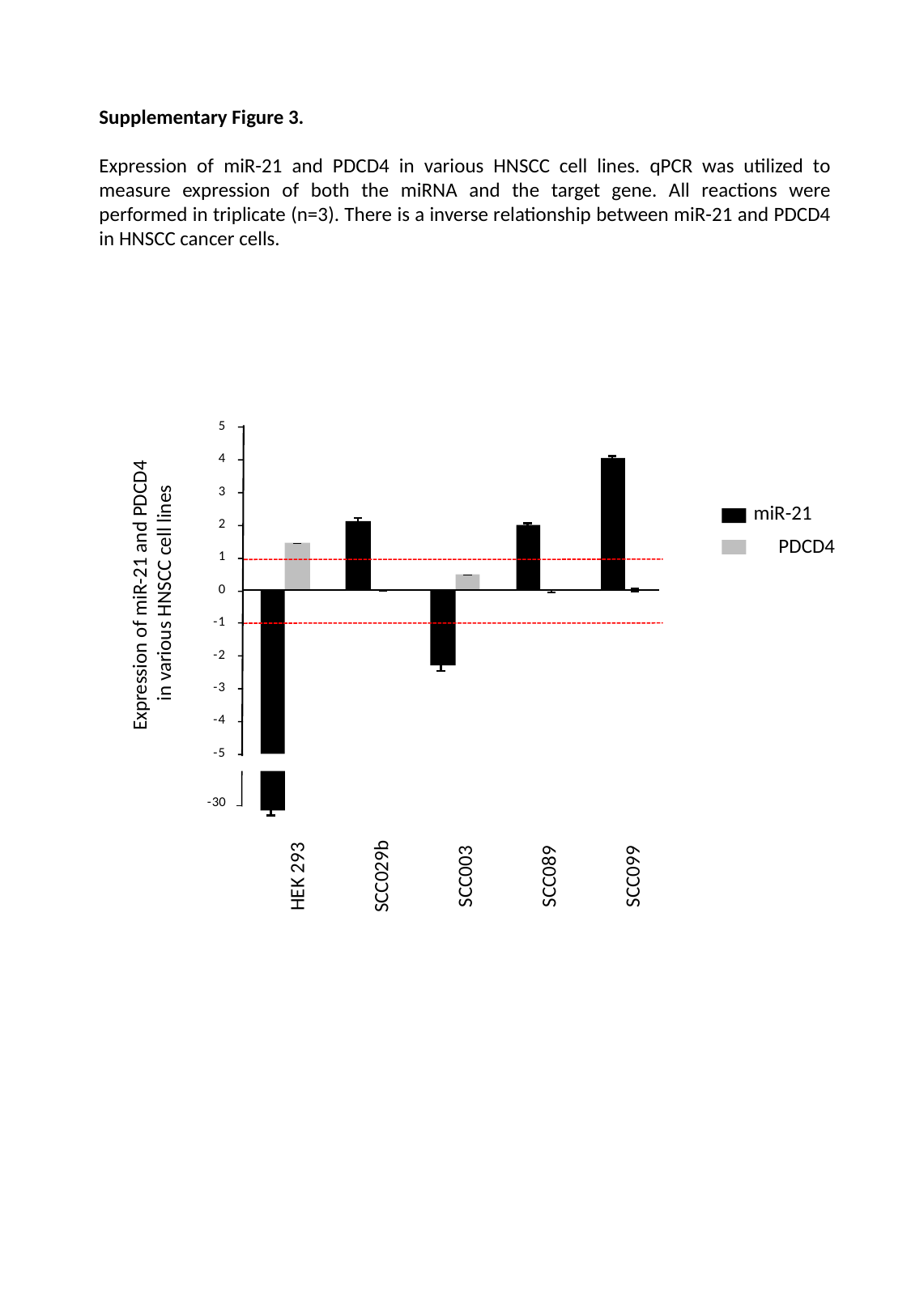

Supplementary Figure 3.
Expression of miR-21 and PDCD4 in various HNSCC cell lines. qPCR was utilized to measure expression of both the miRNA and the target gene. All reactions were performed in triplicate (n=3). There is a inverse relationship between miR-21 and PDCD4 in HNSCC cancer cells.
5
4
3
2
1
0
-
1
-
2
-
3
-
4
-
5
-
30
miR-21
PDCD4
Expression of miR-21 and PDCD4
in various HNSCC cell lines
HEK 293
SCC029b
SCC003
SCC089
SCC099

## Slide 4
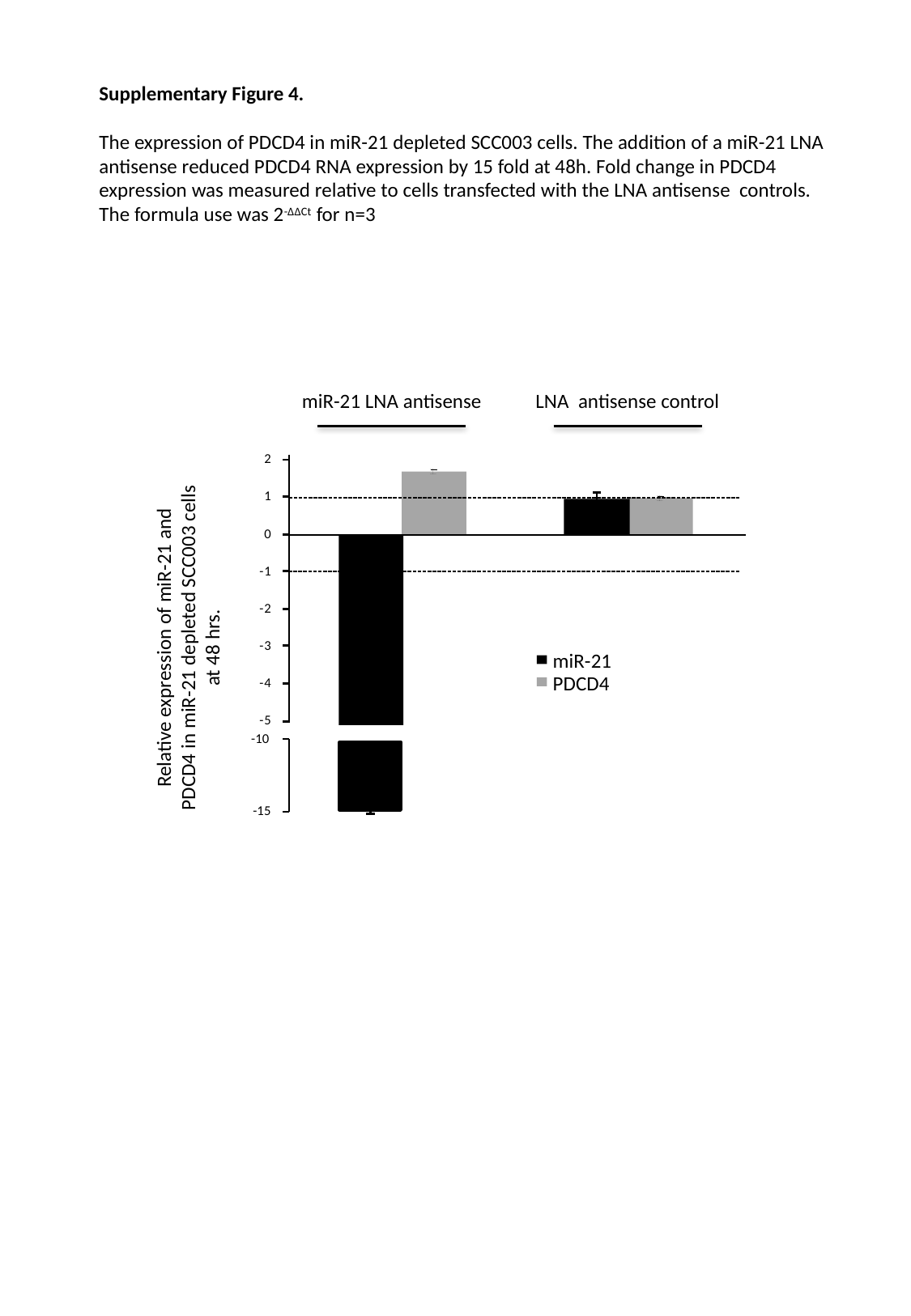

Supplementary Figure 4.
The expression of PDCD4 in miR-21 depleted SCC003 cells. The addition of a miR-21 LNA antisense reduced PDCD4 RNA expression by 15 fold at 48h. Fold change in PDCD4 expression was measured relative to cells transfected with the LNA antisense controls. The formula use was 2-ΔΔCt for n=3
miR-21 LNA antisense
LNA antisense control
2
1
0
-
1
-
2
Relative expression of miR-21 and PDCD4 in miR-21 depleted SCC003 cells at 48 hrs.
-
3
miR-21
PDCD4
-
4
-
5
-10
-15

## Slide 5
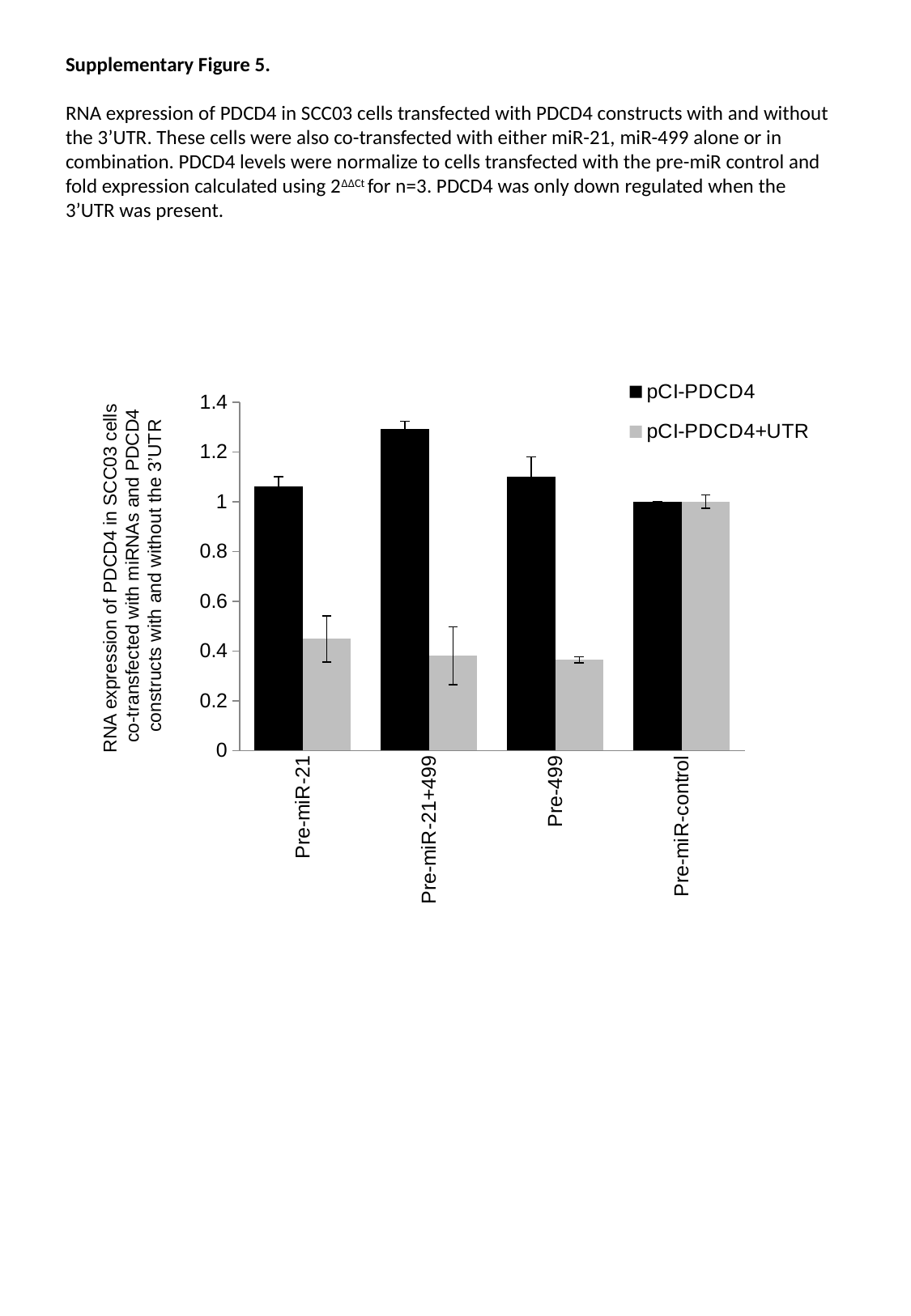

Supplementary Figure 5.
RNA expression of PDCD4 in SCC03 cells transfected with PDCD4 constructs with and without the 3’UTR. These cells were also co-transfected with either miR-21, miR-499 alone or in combination. PDCD4 levels were normalize to cells transfected with the pre-miR control and fold expression calculated using 2ΔΔCt for n=3. PDCD4 was only down regulated when the 3’UTR was present.
### Chart
| Category | pCI-PDCD4 | pCI-PDCD4+UTR |
|---|---|---|
| Pre-miR-21 | 1.062226676631957 | 0.448476252692127 |
| Pre-miR-21+499 | 1.291813677118902 | 0.380607659398211 |
| Pre-499 | 1.101802010073457 | 0.364334747468267 |
| Pre-miR-control | 1.0 | 1.0 |RNA expression of PDCD4 in SCC03 cells co-transfected with miRNAs and PDCD4 constructs with and without the 3’UTR

## Slide 6
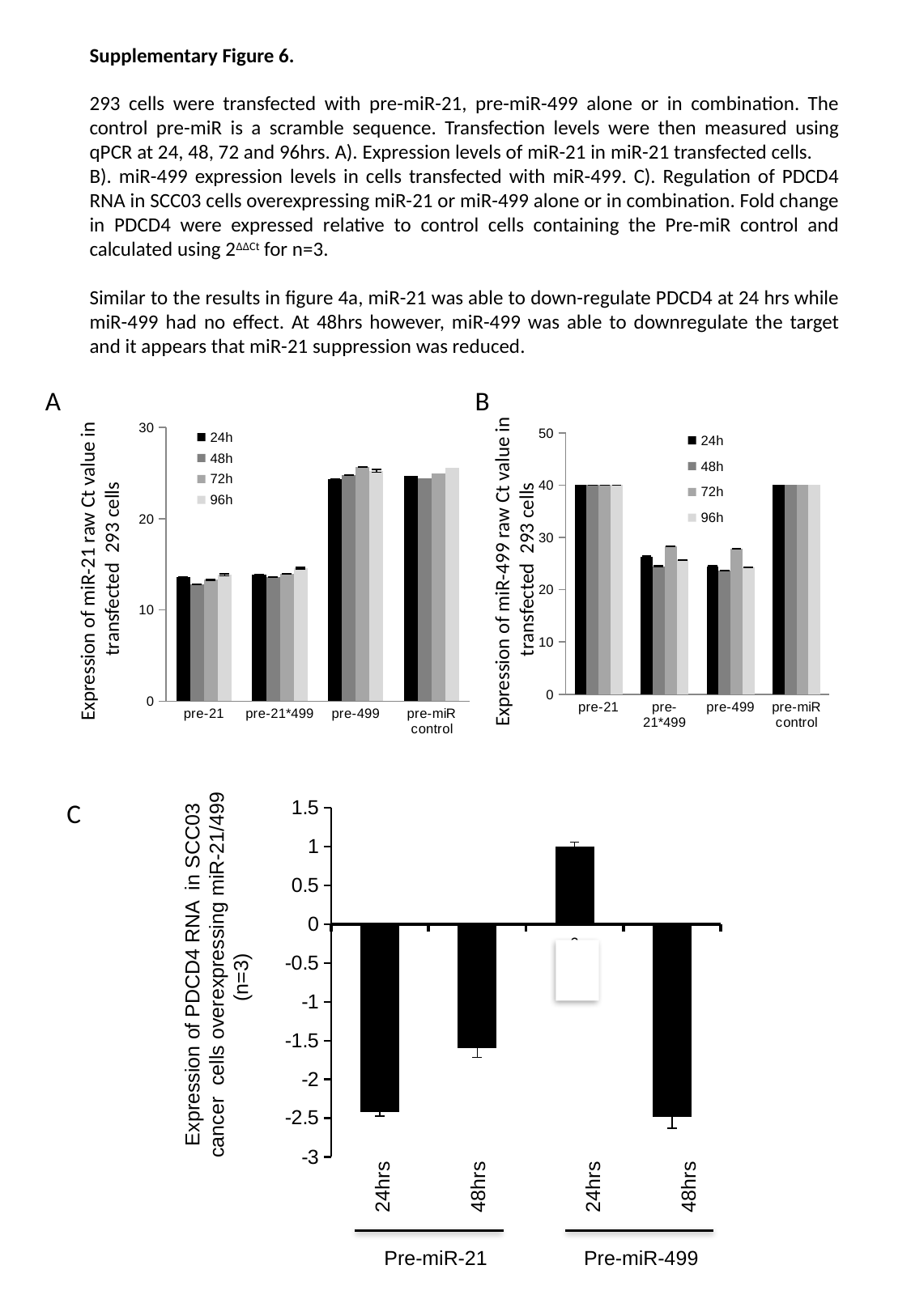

Supplementary Figure 6.
293 cells were transfected with pre-miR-21, pre-miR-499 alone or in combination. The control pre-miR is a scramble sequence. Transfection levels were then measured using qPCR at 24, 48, 72 and 96hrs. A). Expression levels of miR-21 in miR-21 transfected cells.
B). miR-499 expression levels in cells transfected with miR-499. C). Regulation of PDCD4 RNA in SCC03 cells overexpressing miR-21 or miR-499 alone or in combination. Fold change in PDCD4 were expressed relative to control cells containing the Pre-miR control and calculated using 2ΔΔCt for n=3.
Similar to the results in figure 4a, miR-21 was able to down-regulate PDCD4 at 24 hrs while miR-499 had no effect. At 48hrs however, miR-499 was able to downregulate the target and it appears that miR-21 suppression was reduced.
A
B
### Chart
| Category | | | | |
|---|---|---|---|---|
| pre-21 | 13.596453 | 12.784455 | 13.293341 | 13.85341167449952 |
| pre-21*499 | 13.87000600000004 | 13.584378 | 13.939323 | 14.58311939239502 |
| pre-499 | 24.33561899999999 | 24.755665 | 25.64241999999988 | 25.22714805603027 |
| pre-miR control | 24.711527 | 24.45925099999999 | 24.96866799999988 | 25.56114959716772 |
### Chart
| Category | | | | |
|---|---|---|---|---|
| pre-21 | 40.0 | 40.0 | 40.0 | 40.0 |
| pre-21*499 | 26.312422 | 24.501846 | 28.34624099999992 | 25.62152099609375 |
| pre-499 | 24.3570700000001 | 23.64720000000001 | 27.870562 | 24.20674324035625 |
| pre-miR control | 40.0 | 40.0 | 40.0 | 40.0 |Expression of miR-21 raw Ct value in
 transfected 293 cells
Expression of miR-499 raw Ct value in
 transfected 293 cells
C
### Chart
| Category | |
|---|---|Expression of PDCD4 RNA in SCC03 cancer cells overexpressing miR-21/499 (n=3)
24hrs
48hrs
Pre-miR-21
24hrs
48hrs
Pre-miR-499
