# Supplementary material for: Regulation of the tumour suppressor PDCD4 by miR-499 and miR-21 in oropharyngeal cancers
Source: BMC Cancer. 2016 Feb 11;16:86. doi: 10.1186/s12885-016-2109-4 (PMC4750294; doi:10.1186/s12885-016-2109-4)
Supplement: Additional file 2: — Patient Data. (PPTX 73 kb) [file 12885_2016_2109_MOESM2_ESM.pptx]

## Slide 1
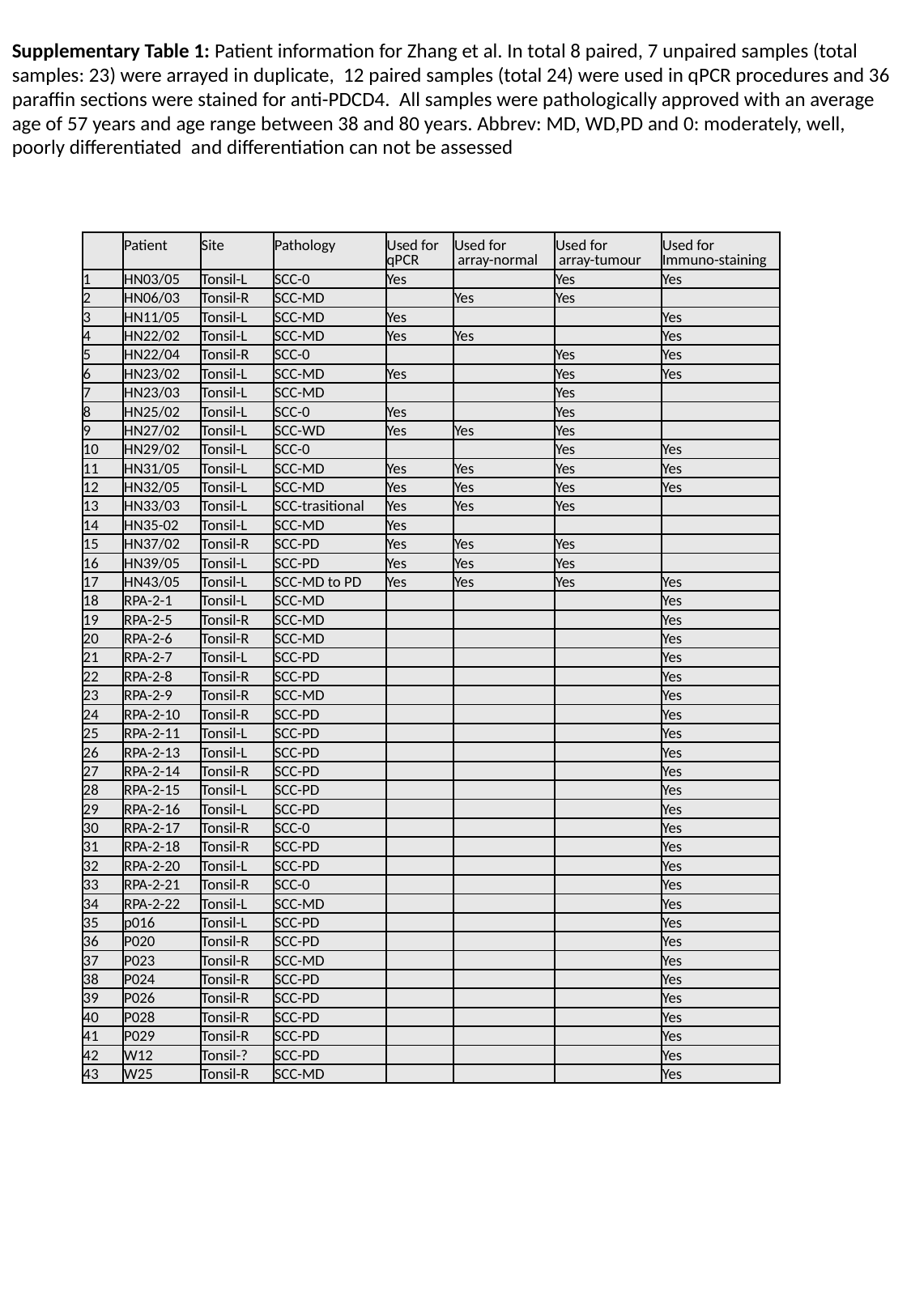

Supplementary Table 1: Patient information for Zhang et al. In total 8 paired, 7 unpaired samples (total samples: 23) were arrayed in duplicate, 12 paired samples (total 24) were used in qPCR procedures and 36 paraffin sections were stained for anti-PDCD4. All samples were pathologically approved with an average age of 57 years and age range between 38 and 80 years. Abbrev: MD, WD,PD and 0: moderately, well, poorly differentiated and differentiation can not be assessed
| | Patient | Site | Pathology | Used for qPCR | Used for array-normal | Used for array-tumour | Used for Immuno-staining |
| --- | --- | --- | --- | --- | --- | --- | --- |
| 1 | HN03/05 | Tonsil-L | SCC-0 | Yes | | Yes | Yes |
| 2 | HN06/03 | Tonsil-R | SCC-MD | | Yes | Yes | |
| 3 | HN11/05 | Tonsil-L | SCC-MD | Yes | | | Yes |
| 4 | HN22/02 | Tonsil-L | SCC-MD | Yes | Yes | | Yes |
| 5 | HN22/04 | Tonsil-R | SCC-0 | | | Yes | Yes |
| 6 | HN23/02 | Tonsil-L | SCC-MD | Yes | | Yes | Yes |
| 7 | HN23/03 | Tonsil-L | SCC-MD | | | Yes | |
| 8 | HN25/02 | Tonsil-L | SCC-0 | Yes | | Yes | |
| 9 | HN27/02 | Tonsil-L | SCC-WD | Yes | Yes | Yes | |
| 10 | HN29/02 | Tonsil-L | SCC-0 | | | Yes | Yes |
| 11 | HN31/05 | Tonsil-L | SCC-MD | Yes | Yes | Yes | Yes |
| 12 | HN32/05 | Tonsil-L | SCC-MD | Yes | Yes | Yes | Yes |
| 13 | HN33/03 | Tonsil-L | SCC-trasitional | Yes | Yes | Yes | |
| 14 | HN35-02 | Tonsil-L | SCC-MD | Yes | | | |
| 15 | HN37/02 | Tonsil-R | SCC-PD | Yes | Yes | Yes | |
| 16 | HN39/05 | Tonsil-L | SCC-PD | Yes | Yes | Yes | |
| 17 | HN43/05 | Tonsil-L | SCC-MD to PD | Yes | Yes | Yes | Yes |
| 18 | RPA-2-1 | Tonsil-L | SCC-MD | | | | Yes |
| 19 | RPA-2-5 | Tonsil-R | SCC-MD | | | | Yes |
| 20 | RPA-2-6 | Tonsil-R | SCC-MD | | | | Yes |
| 21 | RPA-2-7 | Tonsil-L | SCC-PD | | | | Yes |
| 22 | RPA-2-8 | Tonsil-R | SCC-PD | | | | Yes |
| 23 | RPA-2-9 | Tonsil-R | SCC-MD | | | | Yes |
| 24 | RPA-2-10 | Tonsil-R | SCC-PD | | | | Yes |
| 25 | RPA-2-11 | Tonsil-L | SCC-PD | | | | Yes |
| 26 | RPA-2-13 | Tonsil-L | SCC-PD | | | | Yes |
| 27 | RPA-2-14 | Tonsil-R | SCC-PD | | | | Yes |
| 28 | RPA-2-15 | Tonsil-L | SCC-PD | | | | Yes |
| 29 | RPA-2-16 | Tonsil-L | SCC-PD | | | | Yes |
| 30 | RPA-2-17 | Tonsil-R | SCC-0 | | | | Yes |
| 31 | RPA-2-18 | Tonsil-R | SCC-PD | | | | Yes |
| 32 | RPA-2-20 | Tonsil-L | SCC-PD | | | | Yes |
| 33 | RPA-2-21 | Tonsil-R | SCC-0 | | | | Yes |
| 34 | RPA-2-22 | Tonsil-L | SCC-MD | | | | Yes |
| 35 | p016 | Tonsil-L | SCC-PD | | | | Yes |
| 36 | P020 | Tonsil-R | SCC-PD | | | | Yes |
| 37 | P023 | Tonsil-R | SCC-MD | | | | Yes |
| 38 | P024 | Tonsil-R | SCC-PD | | | | Yes |
| 39 | P026 | Tonsil-R | SCC-PD | | | | Yes |
| 40 | P028 | Tonsil-R | SCC-PD | | | | Yes |
| 41 | P029 | Tonsil-R | SCC-PD | | | | Yes |
| 42 | W12 | Tonsil-? | SCC-PD | | | | Yes |
| 43 | W25 | Tonsil-R | SCC-MD | | | | Yes |

## Slide 2
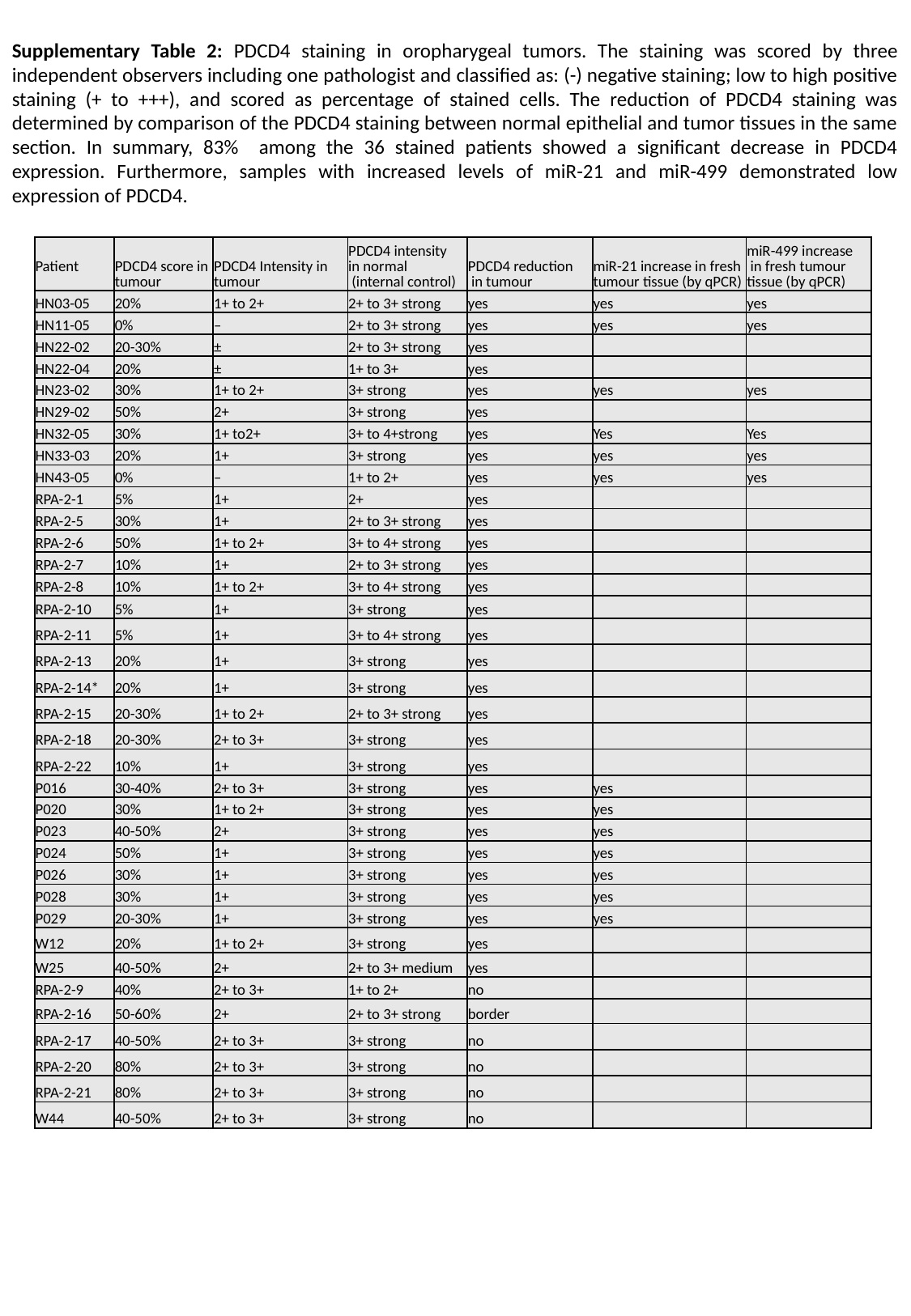

Supplementary Table 2: PDCD4 staining in oropharygeal tumors. The staining was scored by three independent observers including one pathologist and classified as: (-) negative staining; low to high positive staining (+ to +++), and scored as percentage of stained cells. The reduction of PDCD4 staining was determined by comparison of the PDCD4 staining between normal epithelial and tumor tissues in the same section. In summary, 83% among the 36 stained patients showed a significant decrease in PDCD4 expression. Furthermore, samples with increased levels of miR-21 and miR-499 demonstrated low expression of PDCD4.
| Patient | PDCD4 score in tumour | PDCD4 Intensity in tumour | PDCD4 intensity in normal (internal control) | PDCD4 reduction in tumour | miR-21 increase in fresh tumour tissue (by qPCR) | miR-499 increase in fresh tumour tissue (by qPCR) |
| --- | --- | --- | --- | --- | --- | --- |
| HN03-05 | 20% | 1+ to 2+ | 2+ to 3+ strong | yes | yes | yes |
| HN11-05 | 0% | – | 2+ to 3+ strong | yes | yes | yes |
| HN22-02 | 20-30% | ± | 2+ to 3+ strong | yes | | |
| HN22-04 | 20% | ± | 1+ to 3+ | yes | | |
| HN23-02 | 30% | 1+ to 2+ | 3+ strong | yes | yes | yes |
| HN29-02 | 50% | 2+ | 3+ strong | yes | | |
| HN32-05 | 30% | 1+ to2+ | 3+ to 4+strong | yes | Yes | Yes |
| HN33-03 | 20% | 1+ | 3+ strong | yes | yes | yes |
| HN43-05 | 0% | – | 1+ to 2+ | yes | yes | yes |
| RPA-2-1 | 5% | 1+ | 2+ | yes | | |
| RPA-2-5 | 30% | 1+ | 2+ to 3+ strong | yes | | |
| RPA-2-6 | 50% | 1+ to 2+ | 3+ to 4+ strong | yes | | |
| RPA-2-7 | 10% | 1+ | 2+ to 3+ strong | yes | | |
| RPA-2-8 | 10% | 1+ to 2+ | 3+ to 4+ strong | yes | | |
| RPA-2-10 | 5% | 1+ | 3+ strong | yes | | |
| RPA-2-11 | 5% | 1+ | 3+ to 4+ strong | yes | | |
| RPA-2-13 | 20% | 1+ | 3+ strong | yes | | |
| RPA-2-14\* | 20% | 1+ | 3+ strong | yes | | |
| RPA-2-15 | 20-30% | 1+ to 2+ | 2+ to 3+ strong | yes | | |
| RPA-2-18 | 20-30% | 2+ to 3+ | 3+ strong | yes | | |
| RPA-2-22 | 10% | 1+ | 3+ strong | yes | | |
| P016 | 30-40% | 2+ to 3+ | 3+ strong | yes | yes | |
| P020 | 30% | 1+ to 2+ | 3+ strong | yes | yes | |
| P023 | 40-50% | 2+ | 3+ strong | yes | yes | |
| P024 | 50% | 1+ | 3+ strong | yes | yes | |
| P026 | 30% | 1+ | 3+ strong | yes | yes | |
| P028 | 30% | 1+ | 3+ strong | yes | yes | |
| P029 | 20-30% | 1+ | 3+ strong | yes | yes | |
| W12 | 20% | 1+ to 2+ | 3+ strong | yes | | |
| W25 | 40-50% | 2+ | 2+ to 3+ medium | yes | | |
| RPA-2-9 | 40% | 2+ to 3+ | 1+ to 2+ | no | | |
| RPA-2-16 | 50-60% | 2+ | 2+ to 3+ strong | border | | |
| RPA-2-17 | 40-50% | 2+ to 3+ | 3+ strong | no | | |
| RPA-2-20 | 80% | 2+ to 3+ | 3+ strong | no | | |
| RPA-2-21 | 80% | 2+ to 3+ | 3+ strong | no | | |
| W44 | 40-50% | 2+ to 3+ | 3+ strong | no | | |
